# Periodontitis and pulmonary function: a Mendelian randomization study

**DOI:** 10.1007/s00784-021-04000-9

**Published:** 2021-05-27

**Authors:** Sebastian-Edgar Baumeister, Michael Nolde, Birte Holtfreter, Hansjörg Baurecht, Sven Gläser, Thomas Kocher, Benjamin Ehmke

**Affiliations:** 1 Institute of Health Services Research in Dentistry, University of Münster, Albert-Schweitzer-Campus 1 48149, Münster, Germany; 2 Chair of Epidemiology, University of Augsburg, Augsburg, Germany; 3 Unit of Periodontology, Department of Restorative Dentistry, Periodontology, Endodontology, and Preventive and Pediatric Dentistry, University Medicine Greifswald, Greifswald, Germany; 4 Department of Epidemiology and Preventive Medicine, University of Regensburg, Regensburg, Germany; 5 Department of Internal Medicine B Cardiology, Intensive Care, Pulmonary Medicine and Infectious Diseases, University Medicine Greifswald, Greifswald, Germany; 6 Vivantes Klinikum Neukölln Und Spandau, Klinik Für Innere Medizin - Pneumologie Und Infektiologie, Berlin, Germany; 7 Clinic for Periodontology and Conservative Dentistry, University of Münster, Münster, Germany

**Keywords:** Periodontitis, Lung function, Mendelian randomization

## Abstract

**Objectives:**

Observational research suggests that periodontitis affects pulmonary function; however, observational studies are subject to confounding and reverse causation, making causal inference and the direction of these associations difficult. We used Mendelian randomization (MR) to assess the potential causal association between genetic liability to periodontitis and pulmonary function.

**Materials and methods:**

We used six single-nucleotide polymorphisms (SNPs) associated with periodontitis (*P* < 5 × 10^−6^) from a genome-wide association study (GWAS) of 17,353 European descent periodontitis cases and 28,210 controls from the GeneLifestyle Interactions in Dental Endpoints consortium and the UK Biobank, and related these to SNPs from a lung function GWAS including 79,055 study participants of the SpiroMeta Consortium.

**Results:**

MR analysis suggested no effect of periodontitis on the ratio of forced expiratory volume in one second to lower forced vital capacity (standard deviation increment in outcome per doubling of the odds of the exposure (95% confidence interval) =  − 0.004 (− 0.028; 0.020)). Replication analysis using genetic instruments from two different GWAS and sensitivity analyses to address potential pleiotropy led to no substantial changes in estimates.

**Conclusions:**

Collectively, these findings do not support a relationship between genetic liability for periodontitis and pulmonary function.

**Clinical relevance:**

Periodontitis does not seem to be a risk factor for worsening of pulmonary function.

Periodontitis is a chronic, bacteria-initiated inflammatory disease, affecting ~ 50% of adults, with 11.2% suffering from severe periodontitis [1]. Chronic obstructive pulmonary disease (COPD) is a disease with a growing global burden. It is characterized by persistent symptoms and progressive airflow limitations diagnosed by lung function testing. As COPD and periodontitis are both characterized by neutrophilic inflammation with subsequent proteolytic destruction of connective tissue, it has been proposed that they share common pathophysiological processes. Periodontitis has been linked to a higher risk of COPD and worsening of pulmonary function in previous observational research [2, 3]. The latest systematic review identified seven cross-sectional and prospective studies and reported a pooled, confounder-adjusted odds ratio of 1.78 (95% confidence interval (CI): 1.04; 3.05) for COPD [2]. Proposed pathways by which periodontitis might influence pulmonary function are by mechanical aspiration of oral contents into the respiratory tree, the overspill of locally produced inflammatory mediators into the systemic circulation or oral or lung-derived bacteremia activating an acute-phase response, and reactive oxygen species and cytokine release by systemic neutrophils at distant sites [4, 5].

Yet, the observed association between periodontitis and pulmonary function could also be due to confounding, such as tobacco smoking [5], which is difficult to control when relying on observational study designs. One approach to account for observational bias and strengthen causal inference is the method of Mendelian randomization (MR), a form of instrumental variable analysis [6]. The MR method diminishes confounding by environmental factors because alleles are randomly allocated when passed from parents to offspring at conception and avoids reverse causation because disease cannot affect genotype. In MR, the instrument comprises one or more genetic variants that are robustly associated with the exposure of interest. The most widely adopted approach is to rely on inferences from single-nucleotide polymorphisms (SNPs) identified through genome-wide association studies (GWAS).

## Methods

We performed two-sample MR analysis using summary statistics from the largest available GWAS on periodontitis and pulmonary function [7, 8]. We used 6 SNPs associated with periodontitis (*P* < 5 × 10^−6^), after accounting for linkage disequilibrium (r^2^ = 0.001, window size = 10 mB), as instruments from GWAS of 17,353 European decent periodontitis cases and 28,210 controls from the GeneLifestyle Interactions in Dental Endpoints consortium and UK Biobank [8], and related these to SNP-lung function associations from GWAS of 79,055 European decent study participants of the SpiroMeta Consortium [7]. After data harmonization, we calculated Wald ratios by dividing the linear regression coefficients for forced expiratory volume in one second (FEV_1_), lower forced vital capacity (FVC), and FEV_1_/FVC ratio by the corresponding log OR of the same SNP in the GWAS for periodontitis; and obtained standard errors by the delta method. Wald ratios were pooled using the primary multiplicative random effects inverse-variance-weighted (IVW) analyses [9]. We transformed the effect estimates of the binary exposure to reflect doubling in the odds of the exposure on the per one standard deviation (1-SD) increment of the outcome [10]. MR rests on the assumptions that the instrument is robustly associated with the exposure (I), is not associated with confounders (II), and is not associated with the outcome other than via its association with the exposure (III). If the instrument-exposure association is weak, this might reduce the plausibility of assumption I. Violations of assumptions II and III can occur through horizontal pleiotropy, whereby the instruments exert an effect on the outcome independent of the exposure. We used more liberal SNP selection thresholds (*P* < 5 × 10^−4^, *P* < 5 × 10^−5^) to strengthen the instrument and applied weak instrument analyses [6, 9, 11]. We investigated pleiotropy by searching for previously reported associations of instruments with tobacco smoking in PhenoScanner (www.phenoscanner.medschl.cam.ac.uk/) and the GWAS Catalog (www.ebi.ac.uk/gwas/); investigated the Cochran Q heterogeneity test, the I^2^ statistic, and the MR-Egger intercept test; performed leave-one-SNP-out analysis; and applied a suite of pleiotropy-robust methods (weighted median, robust adjusted profile score, radial regression, MR‐pleiotropy residual sum and outlier) [6, 12]. We used two additional GWAS [13, 14] of aggressive and chronic periodontitis to identify instruments in replication analyses and adopted a negative control outcome approach [15]. Height at age 10 years served as a negative control. SNP-outcome associations for height at age 10 years were taken from the Neale 2017 UK Biobank phenome-wide GWAS included in mrbase.org (access date 2021 February 11). As the periodontitis age of onset is typically after puberty, if our instruments affect lung function solely through periodontitis phenotypes, we expect to find no effect on pre-puberty height.

## Results

**Fig. 1:**
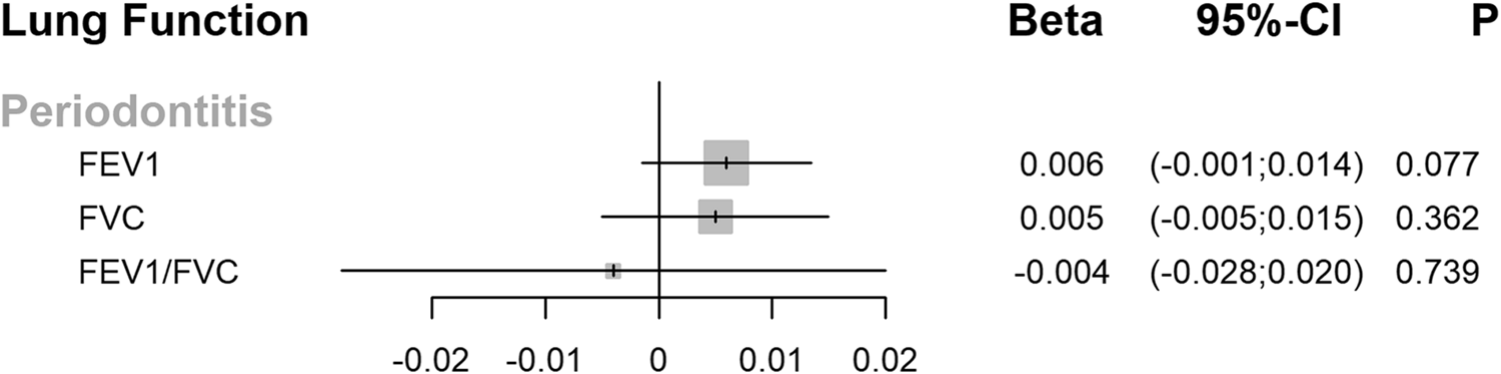
Per one standard deviation change (beta) in forced expiratory volume in 1 s (FEV1), forced vital capacity (FVC), and FEV1/FVC ratio associated with doubling in the prevalence of genetic liability to periodontitis using the inverse-variance-weighted method. *CI*, confidence interval; *P*, *P* value

The IVW analysis did not suggest an effect of genetic liability to periodontitis on FEV_1_, FVC, and FEV_1_/FVC (Fig. 1). For example, a 1-SD of the FEV_1_/FVC ratio decreased by − 0.004 (95% CI: − 0.028; 0.020) per doubling in the odds of periodontitis. The primary analyses were supported when using more liberal SNP selection thresholds and in weak instrument analyses. We found no previously reported associations of genetic variants with tobacco smoking. The IVW estimates were consistent with estimates from robust methods. There was minimal heterogeneity between Wald ratios, and the MR-Egger intercept analyses did not indicate directional pleiotropy. IVW leave-one-out analysis did not identify any leverage points with high influence. In replication analysis, when summary data from two different GWAS of periodontitis were used, effect sizes were similar to the primary analysis. In negative control outcome analysis, the association (IVW Beta = 0.009, 95% CI: − 0.001; 0.003) with height at 10 years provided additional reassurance for the robustness of the primary analysis.

## Discussion

The association between periodontitis and pulmonary function seen in previous observational studies was not replicated when applying MR and could have been due to unobserved environmental confounding or selection bias [5]. The study had limitations. The phenotypic variance explained by the 6 SNPs in primary analysis was only 0.8%; however, the minimum F statistic was 22 and did not raise concerns of weak instrument bias. Additional weak instrument analyses provided higher statistical power and also did not suggest an association. The biologic mechanisms of the selected SNPs are unknown; however, sensitivity analyses failed to find evidence for horizontal pleiotropy. The primary periodontitis GWAS [8] used a broadly defined phenotype including the Centers for Disease Control and Prevention/American Academy of Periodontology definition of periodontitis or participant-reported diagnosis of periodontitis, which might have introduced measurement error. However, classical measurement error in the exposure does not affect asymptotic estimates from instrumental variable analysis. Also, replication analysis using SNPs from two GWAS [13, 14] of clinically defined periodontitis yielded similar point estimates. Furthermore, the phenotype was binary so we could not assess potential dose-dependent changes in pulmonary function. Finally, the SNP effect estimates were obtained from European studies, and caution is warranted before generalizing findings to other populations.

Collectively, our data provided preliminary evidence that genetically proxied long-term exposure to periodontitis does not worsen pulmonary function. We emphasize the importance of triangulating multiple lines of experimental and observational research to strengthen causal inference [16].
